# Role of PXR in Hepatic Cancer: Its Influences on Liver Detoxification Capacity and Cancer Progression

**DOI:** 10.1371/journal.pone.0164087

**Published:** 2016-10-19

**Authors:** Deepak Kotiya, Bharti Jaiswal, Sampa Ghose, Rachna Kaul, Kasturi Datta, Rakesh K. Tyagi

**Affiliations:** 1 Special Centre for Molecular Medicine, Jawaharlal Nehru University, New Delhi, India; 2 School of Environmental Sciences, Jawaharlal Nehru University, New Delhi, India; University of Navarra School of Medicine and Center for Applied Medical Research (CIMA), SPAIN

## Abstract

The role of nuclear receptor PXR in detoxification and clearance of xenobiotics and endobiotics is well-established. However, its projected role in hepatic cancer is rather illusive where its expression is reported altered in different cancers depending on the tissue-type and microenvironment. The expression of PXR, its target genes and their biological or clinical significance have not been examined in hepatic cancer. In the present study, by generating DEN-induced hepatic cancer in mice, we report that the expression of PXR and its target genes CYP3A11 and GSTa2 are down-regulated implying impairment of hepatic detoxification capacity. A higher state of inflammation was observed in liver cancer tissues as evident from upregulation of inflammatory cytokines IL-6 and TNF-α along with NF-κB and STAT3. Our data in mouse model suggested a negative correlation between down-regulation of PXR and its target genes with that of higher expression of inflammatory proteins (like IL-6, TNF-α, NF-κB). In conjunction, our findings with relevant cell culture based assays showed that higher expression of PXR is involved in reduction of tumorigenic potential in hepatic cancer. Overall, the findings suggest that inflammation influences the expression of hepatic proteins important in drug metabolism while higher PXR level reduces tumorigenic potential in hepatic cancer.

## Introduction

Pregnane and Xenobiotic Receptor (PXR), acts as a ‘master-regulator’ of expression of components of the detoxification machinery thereby defending the body from the toxic chemical insults [1]. The protective role of PXR is executed by regulating phase I (Cyp3a11 etc.), phase II (Gsta2 etc.) drug metabolizing enzymes and drug transporters (MDR1, MRP3 etc.). PXR is primarily expressed in liver and intestine where maximum detoxification of noxious compounds occurs. However, its lower expression is also detected in other tissues like breast, heart, stomach, adrenal gland, bone marrow, colon, blood-brain barrier, osteoclasts, placenta, ovary, peripheral blood monocytes and uterus [2]. In recent years, apart from its role in endobiotic and xenobiotic metabolism, the functions of PXR have been extended to inflammation and cancer. PXR has been shown to express in various cancers such as colon [3–6], breast [7], prostate [8, 9], endometrial [10], esophageal [11], ovarian [12] and bone cancers [13]. PXR is reported to be overexpressed in breast [7], esophageal [11] and bone [13] cancers. Further, in colon [6] and endometrial cancer [10] a differential expression of PXR is reported, while in prostate [9], cervical [14] and colon [5] cancers down-regulation of PXR is reported. The higher expression of PXR in breast [7], esophageal [11], endometrial [10], prostate [8] and colon [3] cancers has been shown to be associated with higher expression of drug metabolizing enzymes and drug transporters, which leads to multidrug resistance and favours progression of cancer. Whereas, in colon [15] and cervical [14] cancers PXR was observed to have protective role (e.g. inhibits cell proliferation and tumourigenicity), suggesting its possible role in suppression of these cancers. So, there is ambiguity in the role of PXR in cancer which is evident by its differential expression pattern in different cancers.

Primary liver cancer, mostly hepatocellular carcinoma (HCC), is an example of inflammation-related cancer as more than 90% of HCCs arise in the context of hepatic injury and inflammation [16]. There are some reports which showed that inflammatory cytokines IL-6 cause a marked decrease in PXR and its target genes such as *Mrp2*, *Bsep*, and *Cyp3a11* [17] while, NF-κB and PXR mutually repress each other upon activation [18]. However, no concrete studies in hepatic cancer with expression of PXR and its target genes in correlation with inflammation have been reported.

PXR is reported to control the apoptosis and cell proliferation in cancerous conditions. For example, in breast and colon cancer cell lines, activation and overexpression of PXR inhibited the cell proliferation [5, 19]. Further, in colon tumors PXR expression was low [5]. Conversely, in colon cancer PXR activation down-regulated the expression of pro-apoptotic genes including P53 and BAK1, suggesting that PXR activation prevents induction of apoptosis while, it also sensitizes the cells to oxidative stress, which may have implications in the growth and promotion [20, 21]. In view of the present ambiguity, we have attempted to examine the expression of anti-apoptotic and cell-cycle regulatory genes in hepatic cancer that play a crucial role in survival of the cancer cell.

The *in vivo* study here documents the expression of PXR and its key regulatory enzymes in hepatic cancer. We also examined the key inflammatory proteins and correlated their expression levels with PXR and its target genes in hepatic cancer. Subsequently, to garner further support, we performed cell culture based assays and examined the effect of PXR overexpression on tumorigenic properties of the cells and also at histological level *in vivo* in transgenic mice. Our observations confirmed that higher expression of PXR reduces the onset and progression of cancer properties of a cancer cell. The conclusions were derived from cell culture based assays such as cell migration, cell invasion, cell adhesion, cell-ECM interactions, cell proliferation and anchorage-independent growth in hepatic cancer. Overall, our observations appear to have important implications in the treatment of hepatic cancer where hepatic drug biotransformation, and bioavailability of administered drugs are under the influence of PXR.

## Materials and Methods

### Chemicals

TRI-reagent for RNA isolation, plasmid/DNA transfection reagents Escort III/Lipofectamine, DEN (Di-ethyl-nitrosamine), Dulbecco’s Modified Eagle’s Medium (DMEM) were obtained from Sigma-Aldrich (St. Louis, MO, USA). Fetal Bovine Serum (FBS) was procured from PAN (Germany). SYBR green master mix obtained from GeneX (India), Revert Aid^TM^ H minus first strand cDNA synthesis kit, Fermentas (USA). Polyvinyldifluoridine (PVDF) membrane was procured from MDI (Ambala, India), 24-well Matrigel-coated chamber inserts (Corning, Sigma-Aldrich, USA), skimmed milk powder from Titan Media (India). Antibodies against mouse PXR, RXR-α and β-actin were generated in our laboratory according to the procedure described earlier by Saradhi et al [22]. Anti-mouse IL-6 and anti-mouse TNF-α antibodies were procured from BD, Biosciences (US). Anti-mouse Rel-A (P65) antibody was obtained from Abcam (USA). Anti-mouse CAR, anti-mouse AFP, anti-goat horseradish peroxidase-conjugated secondary antibody were obtained from Santa Cruz Biotechnology (USA). Anti-rabbit horseradish peroxidase-conjugated secondary antibody was obtained from Sigma-Aldrich (St. Louis, MO, USA). All primers were synthesized from Sigma-Aldrich (India). All other general chemicals and reagents used in the study were of analytical grade and procured from different commercial sources.

### Animals and drug treatments

C57BL/6J mouse strain was bred and maintained at the animal house facility of Jawaharlal Nehru University, New Delhi. Two groups of male mice (six animals/group) were maintained under standard laboratory conditions in clean cages with food and water made available *ad libitum*, under 12 h light/dark cycle at 25 ± 2°C. DEN was introduced by intra-peritoneal injection at a dose of 30 mg per kg of body weight in 13 days old C57BL/6J mice pups. At the same time saline (as a vehicle) was introduced into the control mice. Finally, mice were sacrificed during nine months period and livers were isolated and stored in -80°C and liquid nitrogen (-196°C). For animal experiments, ethical clearance was obtained from institutional animal ethical committee Jawaharlal Nehru University, New Delhi, India and specifically approved this study. A neonatal mouse model of C57BL/6J mice strain was used to generate the hepatic cancer as previously described by Femke Heindryckx et al [23].

### Transgenic mice generation

FVB/J male mice of 30 ± 2 days age were used to generate the FLAG-tagged mouse PXR transgenic mice. The FLAG-tagged mouse PXR construct was introduced into the mice by ‘chemiporation and gene transfer in testicular germ cell method’ as described by Suveera Dhup et al. [24] under standard laboratory conditions in transgenic facility of National Institute of Immunology, New Delhi, India.

### Cell culture

The ATCC human liver cell lines (HepG2, Hep3B and Chang liver cells) were obtained either directly from ATCC or from National Center for Cell Science repository (NCCS, Pune, India). Cells were grown in DMEM supplemented with 10% FBS, 100 μg/ml penicillin, 100 μg/ml streptomycin and 0.25 μg/ml amphotericin. The cultures were maintained at 37°C in a humidified incubator in 5% CO_2_ and 95% air atmosphere. Human HepXR (human PXR stably integrated in HepG2), mouse HepXR (mouse PXR stably integrated in HepG2) and HepR21 (human HABP1 stably integrated in HepG2) were maintained under the same conditions as described earlier [22, 25].

### RNA isolation and real-time PCR

Total RNA from cells and tissues was isolated using TRI-reagent according to the manufacturer’s protocol. The concentration of total RNA was determined by reading the O.D. at 260 nm. Purity of RNA was determined by the ratio between the O. D. at 260 nm to 280 nm and O.D at 260 nm to 230 nm. Total RNA (5 μg) was used as template for reverse transcription by using the RevertAid first strand cDNA synthesis kit. Expressions of different genes were assessed in liver tissue samples retrieved from DEN-induced cancerous and saline-treated control mice by real-time PCR using SYBR green master mix. Real-time quantitative PCR was performed with gene-specific primers (Table A in S1 File) in a total reaction volume of 20 μl. PCR conditions were used as an initial single activation step at 95°C for 15 min, was followed by 40 cycles of amplification [consisting of denaturation at 95°C for 30 sec, annealing at Tm (°C) for 1 min and final extension at 72°C for 30 sec], followed by a melt curve analysis (55–60°C, 15 sec, 40 cycles).

A house-keeping gene, mouse GAPDH was simultaneously amplified (201 bp amplicon size) in separate reactions (at 60°C for 30 sec, annealing) which served as an internal control. The Ct values were corrected using corresponding Ct values of GAPDH controls. The comparative threshold method was used for relative quantification of gene expression by following formula: ΔCt = Ct (test gene)–Ct (GAPDH); ΔΔCt (test gene) = ΔCt (test gene in treatment group) – ΔCt (test gene in vehicle group); the fold change of mRNA = 2^-ΔΔCt^, which indicates the mRNA level of the corresponding transcript in relation to that of the control samples.

### Western blot analysis

For western blotting, equal amount of proteins were resolved by 10% SDS-PAGE. Proteins were blotted onto the methanol charged PVDF membrane using wet transfer system. Following transfer, the membrane was blocked with 5% skimmed milk powder dissolved in Tris-buffer saline with 0.1% Tween-20 (TBST) for 1 hr at room temperature and then incubated overnight at 4°C with primary antibody in appropriate dilutions according to the experimental requirement. The PVDF membrane was then washed four times with TBST and incubated for 1 hr with 1:10,000 dilution of horseradish peroxidase-conjugated anti-rabbit secondary antibody or horseradish peroxidase-conjugated anti-goat secondary antibody. Subsequent to three washes with TBST, the antigen-bound antibody complexes were detected using the enhanced chemi-luminescence (ECL) system.

### Cell culture assays

Chang liver, Hep3B, HepG2, human HepXR and mouse HepXR cell lines were propagated and used under specified cell culture based assays.

#### Wound healing assay

Cell lines were culture to 70–80% confluency. Cells were then scratched with a 200 μl of pipette tip to stimulate the wound. The width of the cell-free wounded zone was monitored at 0 hrs, 24 hrs and 48 hrs by capturing images using Olympus microscope and the migration ability of the cells to fill the gap was calculated using Image J software.

#### Cell adhesion assay

As described by Kaul R. et al [25], all cell lines were seeded in triplicate with 4×10^4^ cells per well of 48 well plate. Cells were fixed with 4% paraformaldehyde for 10 minutes and stained with 0.1% (W/V) crystal violets for 20 minutes at 2 hrs, 4 hrs and 6 hrs, followed by solubilization in 1% Triton X 100 for 24 hrs. The adhesion ability of the cells was calculated by taking the reading at 620 nm using plate reader. The results obtained were corrected by the subtraction of the background staining.

#### Colony formation assay

As described by Kaul R. et al. [25] 1% agarose was melted and mixed with an equal volume of 2X DMEM containing 20% FBS to get 0.5% agarose in 10% FBS-DMEM. In each of the 35 mm dish, 1.5 ml of this 0.5% agarose in FBS-DMEM was poured as base agar. The dishes were left at RT to allow the base agar to solidify. For top agar, 0.7% agarose was melted and cooled to 40°C and mixed with equal volume of 2X DMEM. Cells (15,000 cells/ml) were added to 4.5 ml of DMEM-agarose mix at 40°C and 1.5 ml of agarose was added to each triplate plate for each cell line. The assay plates were then incubated at 37°C in humidified incubator for 3 weeks. Subsequently, plates were stained with 0.5 ml of 0.005% (W/V) Crystal violet for about 3–4 hrs followed by washing with PBS. Images were captured by using Sony BionZ X camera. Anchorage-independent growth of the cells was calculated by manually counting the colonies using Microsoft office picture manager software at 200% zoom by choosing area randomly on the plate.

#### Matrigel invasion assay

The cells were seeded onto a 24-well Matrigel-coated chamber inserts. After 36 hrs, cells on the inside of the inserts were removed with cotton tips, and the invaded cells on the outside of the inserts were stained with crystal violate stain followed by washing with PBS. The cells were visualized and counted under the normal light microscope.

### Doubling time

Cells were seeded in 24-well tissue culture plates at a density of 4 × 10^4^ cells per well in complete Dulbecco’s modified Eagle’s medium, and subsequently, the medium was changed after every 48 hours. At specific times over 5 days, relative cell numbers per well were determined by the 3-[4, 5-dimethylthiazol-2-yl]-2, 5-diphenyltetrazolium bromide (MTT) colorimetric assay on a plate reader at a wavelength of 550 nm with a 650-nm reference filter. Relative growth rates, as a function of time (days), were determined by graphical evaluations of the optical density.

### Immunohistochemistry

Immunohistochemistry was performed using standard histochemical procedures according to the protocol from Thermo Scientific. Formalin-fixed, paraffin-embedded sections were deparaffinized. If required, tissues were incubated with endogenous peroxidase. Endogenous peroxidase activity was blocked by incubating the sections for 10 min with 3% hydrogen peroxide and further non-specific background was prevented by using blocker reagent. The sections were incubated overnight with anti-FLAG primary antibody (1:100). After washing with buffer, the sections were incubated by HRP polymer quanto followed by DAB (Diaminobenzidine tetrahydrochloride) as a chromogen.

### Statistical analysis

Experiments were done at least three times in duplicates or triplicates and the values represent the mean ±SE of three separate experiments. The P-value <0.05 represented by a single asterisk (*), P-value <0.005 represented by a double asterisk (**), P-value <0.0005 represented by a triple asterisk (***) were taken as statistically significant values.

## Results

To elucidate the role of PXR in hepatic cancer we first generated hepatic cancer in C57BL/6J mice and examined the expression of PXR and its target genes along with some of the critical inflammatory proteins.

### Expression of PXR, CAR and their heterodimeric partner RXR-α in hepatic cancer tissues

**Fig 1:**
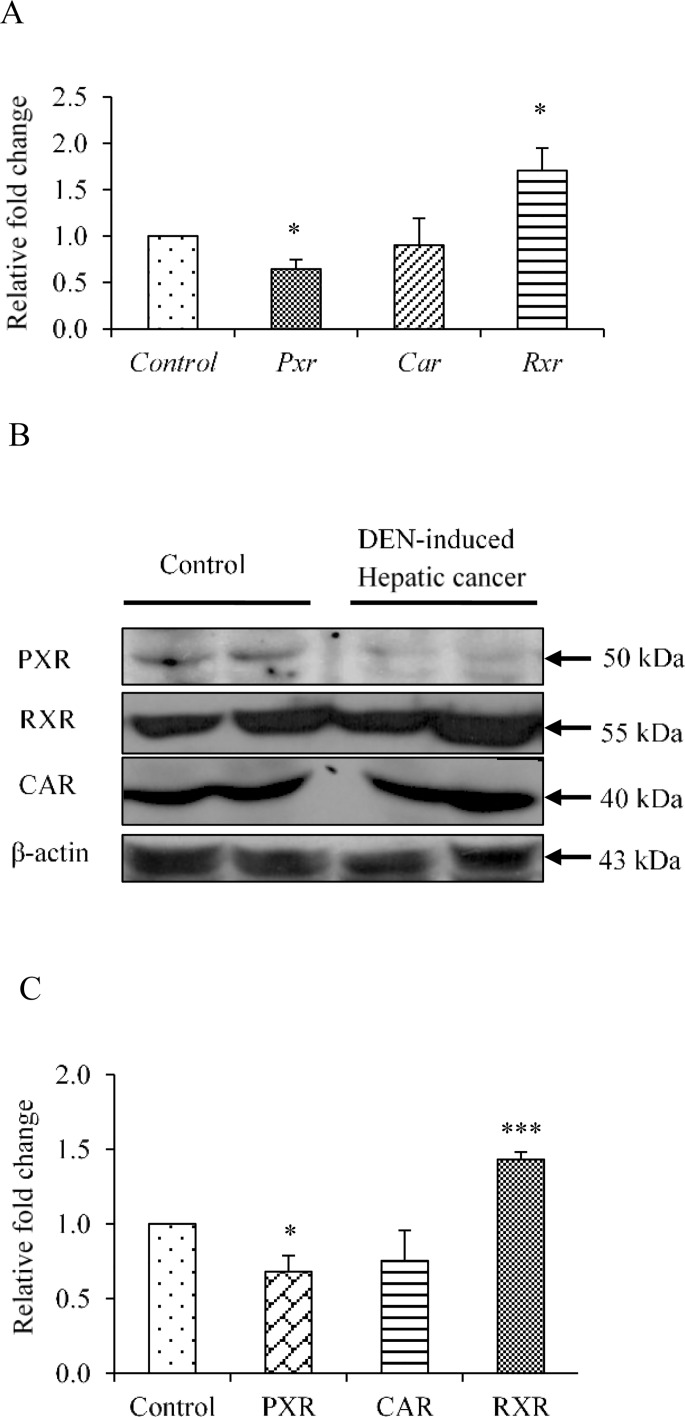
Assessment of expression levels of nuclear receptor PXR, CAR and RXR-α in DEN-induced hepatic cancer. A. Real-time PCR analysis was performed for PXR, CAR and RXR-α genes with the total RNA extracted from mouse liver tissues of saline-treated control and DEN-induced hepatic cancer mice. GAPDH served as an internal control. B. Cell lysate of control and hepatic cancerous mice liver tissues were electrophoresed (50 μg per sample) on a 10% SDS-PAGE. The proteins were transferred onto the methanol-activated PVDF membrane and probed with anti-mouse PXR antibody, anti-mouse CAR antibody and anti-mouse RXR-α antibody. β-Actin antibody served as control; C. The relative endogenous protein expression levels of PXR, CAR and RXR-α in control and DEN-induced hepatic cancer were quantified by densitometry. The experiments were performed with six samples of each control and DEN-induced cancerous mice. The values are represented the mean ±SE. The P-value represents the significance in DEN-induced hepatic cancer mice as compared to the control mice. P-value <0.05 represented by a single asterisk (*), P-value <0.005 represented by a double asterisk (**), P-value <0.0005 represented by a triple asterisk (***).

Hepatic cancer was generated in C57Bl/6J mice by DEN as described under *‘Materials and Methods’*. Hepatic cancer was visually observed by histopathological evaluation (hepatic cord thickening and hepatic portal triad disruption etc.) and by testing for the HCC marker AFP (α-Feto protein), which was >3.00 fold higher at both the transcript as well as protein level when compared to control mice (Figure A in S1 File). We next examined the endogenous expression of PXR, CAR and their heterodimeric partner RXR-α. The expression of PXR was reduced to 0.6 fold both at the transcript (Fig 1A), as well as protein level (Fig 1B and 1C) as compared to control mice. The expression of CAR was reduced to 0.9 fold at the transcript level (Fig 1A) while reduced to 0.7 fold at the protein level (Fig 1B and 1C) respectively in DEN-induced hepatic cancer as compared to control mice. The expression of RXR-α was about 2 fold higher at the transcript level (Fig 1A) while 1.5 fold higher at the protein level (Fig 1B and 1C) respectively in DEN-induced hepatic cancer as compared to control mice. This lower expression of PXR was suggestive of an impaired endobiotic and xenobiotic response in cancerous condition.

### Modulation of representative PXR-regulated genes of phase I, phase II DMEs and drug transporters in hepatic cancer tissues

**Fig 2:**
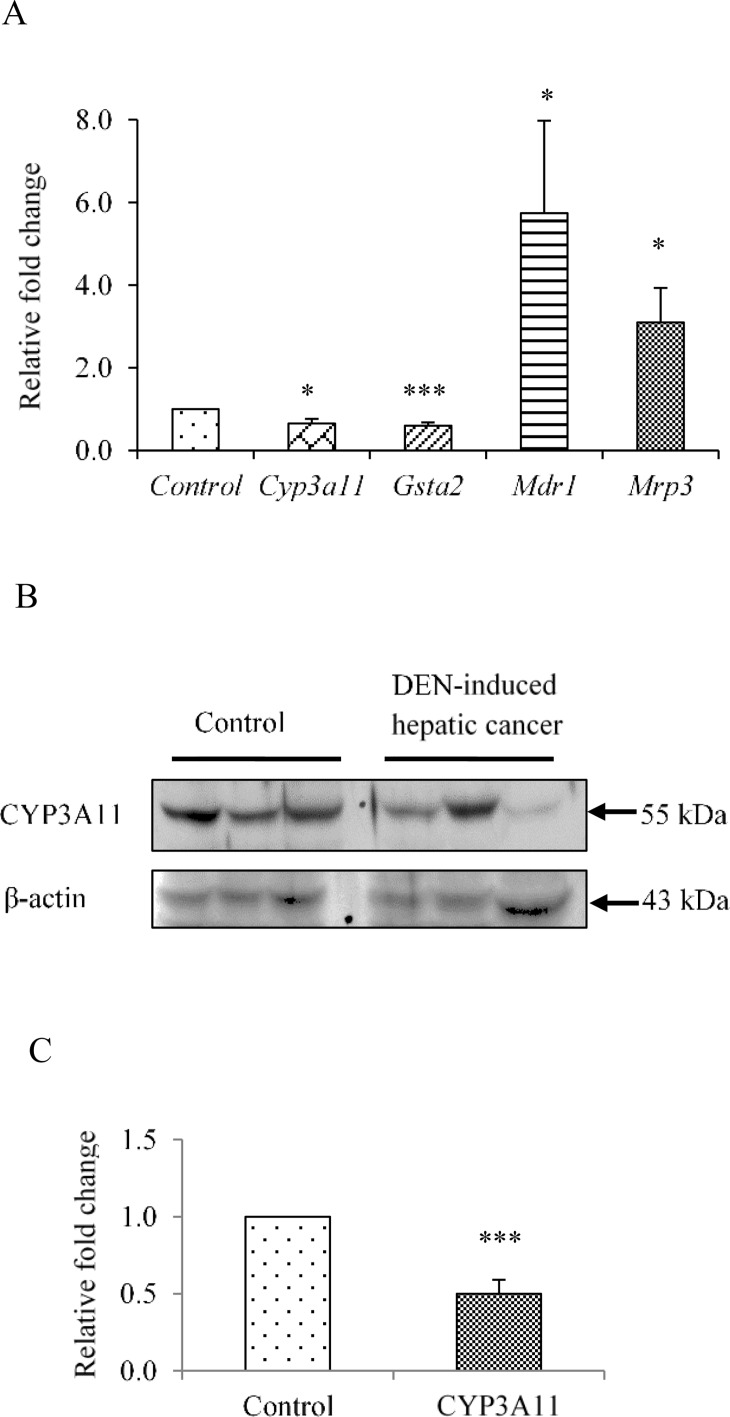
Altered expression of PXR-regulated genes in DEN-induced hepatic cancer. A. Total RNA was extracted from mouse liver tissues of saline-treated control and DEN-induced hepatic cancer mice. Real-time PCR analysis was performed for *Cyp3A11*, *Gsta2* and *Mrp3*. For *Mdr1* semi-quantitative PCR was performed. GAPDH served as an internal control. B. Cell lysates of control and hepatic cancerous mice liver tissues were electrophoresed (50 μg per sample) on a 10% SDS-PAGE. The proteins were transferred onto the methanol-activated PVDF membrane and probed with anti-mouse Cyp3a11 antibody (upper panel). β-Actin antibody served as control (lower panel); C. The relative endogenous Cyp3a11 protein expression in control and DEN-induced hepatic cancer was quantified by densitometry. The experiments were performed with six samples of each control and DEN-induced cancerous mice, and the values are represented as the mean ±SE. The P-value represents the significance in DEN-induced hepatic cancer mice as compared to the control mice. The P-value <0.05 represented by a single asterisk (*), P-value <0.005 represented by a double asterisk (**), P-value <0.0005 represented by a triple asterisk (***).

In view of the results observed above where PXR and CAR expression were lowered in hepatic cancer, it was reasonable to assess if the components of detoxification machinery are also altered. CYP3A11 and GSTa2 are the major enzymes regulated by PXR and belong to phase I and phase II DMEs respectively. We found the expressions of *Cyp3a11* and *Gsta2* were reduced to 0.64 and 0.60 fold respectively at transcript level (Fig 2A), while at protein level CYP3A11 was reduced to 0.49 fold (Fig 2B and 2C) as compared to control mice. Further, we examined the expression of two important PXR-regulated drug transporters *Mdr1* and *Mrp3* which were assessed to be 5.7 and 3.0 fold up-regulated at the transcript level respectively (Fig 2A and Figure B in S1 File) as compared to control mice. Overall, our result suggested that drug metabolizing capacity is altered in hepatic cancer that may result into impaired drug metabolizing capacity.

### Expression of inflammatory proteins in hepatic cancer tissues

**Fig 3:**
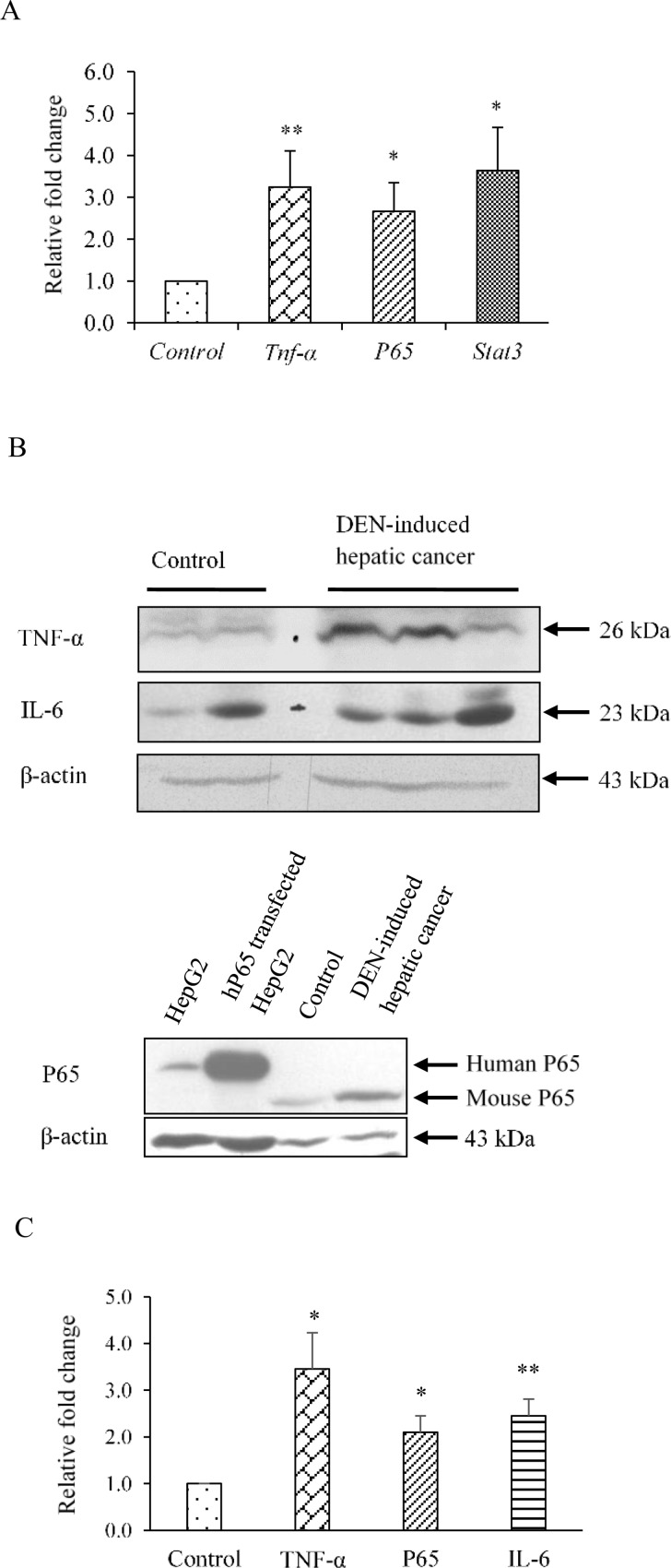
Levels of inflammatory proteins are enhanced in DEN-induced hepatic cancer. A. Real-time PCR analysis was performed for *Tnf-α*, *p65*, and *Stat3* with total RNA extracted from mouse liver tissues of saline-treated control and DEN-induced hepatic cancer mice. GAPDH served as an internal control. B. Cell extracts of control and DEN-induced hepatic cancerous mice liver tissues were electrophoresed (50 μg per sample) on a 10% SDS-PAGE. The proteins were transferred onto the methanol-activated PVDF membrane and probed with anti-mouse TNF-α, anti-mouse P65, and anti-mouse IL-6 antibody. Bands of human and mouse P65 were detected at 65 KDa and 60 KDa respectively. β-Actin antibody served as control; C. The relative endogenous TNF-α, P65 and IL-6 protein expressions in control and DEN-induced hepatic cancer were quantified by densitometry. The experiments were performed with seven samples of each control and DEN-induced cancerous mice, and the values are represented as the mean ±SE. The P-value represents the significance in DEN-induced hepatic cancer mice as compared to the control mice. P-value <0.05 represented by a single asterisk (*), P-value <0.005 represented by a double asterisk (**), P-value <0.0005 represented by a triple asterisk (***).

To further elucidate the reason behind the down-regulation of PXR and PXR-regulated phase I, phase II DMEs we examined the expression of some important inflammatory proteins. IL-6, STAT3, TNF-α and NF-κB are reported to play a central role in inflammation and control the expression of an array of growth factors and cytokines that may involve in hepatic cancer development [26]. Therefore, we examined the expression of all the above four genes in this part of the study. The expression of IL-6 was observed to be 2.4 fold upregulated at the protein level (Fig 3B and 3C) and that for Stat3 about 3.5 fold upregulated at transcript level (Fig 3A) in DEN-induced hepatic cancer as compared to control mice. The expression of TNF-α and P65 (NF-κB) were enhanced to about 3.0 fold and 2.66 fold respectively at transcript level (Fig 3A), while about 3.0 fold and 2.0 fold enhanced respectively at protein level (Fig 3B and 3C) in DEN-induced hepatic cancer as compared to control mice. These results suggest that inflammation may be playing some key role in impaired drug metabolizing capacity in hepatic cancer.

### Assessment of a correlation between the reduced levels of PXR, components of detoxification machinery with inflammatory proteins in hepatic cancer tissues

**Fig 4:**
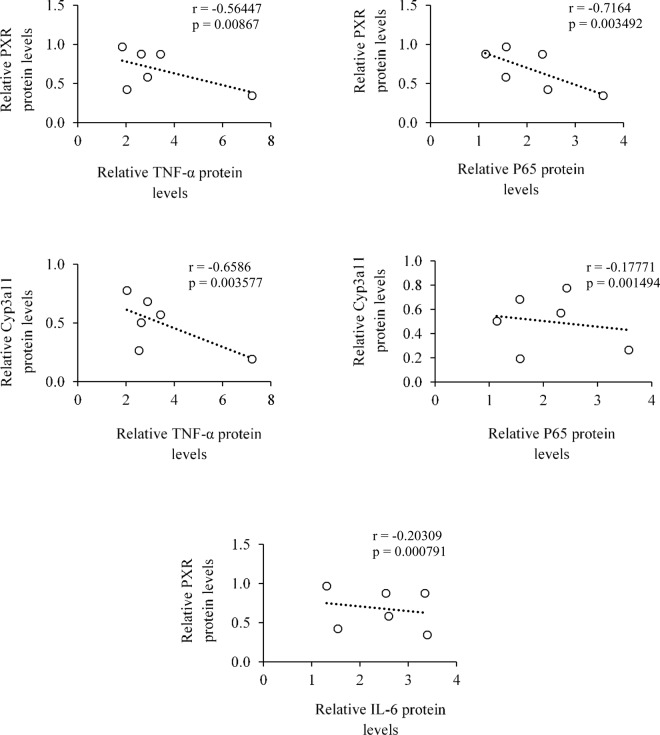
Correlations between the reduced levels of PXR and PXR-regulated CYP3A11 DME with enhanced levels of inflammatory proteins in hepatic cancer. Relative protein levels of TNF-α, P65, and IL-6 were correlated with PXR and CYP3A11 protein levels using Pearson’s correlation coefficient (r) in scattered plot. Respective p-value (p) represents the significance between the correlations.

We successfully correlated the relative transcript and protein levels of TNF-α, P65 and IL-6 with PXR and CYP3A11. We found a negative correlation between TNF-α with PXR and CYP3A11 both at mRNA and protein levels (Fig 4 and Figure C in S1 File). Likewise, a negative correlation between P65 with PXR and CYP3A11 at mRNA and protein levels were observed (Fig 4 and Figure C in S1 File). Similar observation for relative protein levels for cytokine IL-6 with PXR was also observed (Fig 4). Overall, our data suggests that upregulation of TNF-α, P65 and IL-6 may reduce the PXR levels thereby influencing its cellular functions in hepatic cancer tissues.

### Higher expression levels of PXR contribute to reduced tumorigenic potential in hepatic cancer cell lines

We next assessed the involvement of PXR on various metastatic properties of cells such as cell migration, cell invasion, cell adhesion and anchorage-independent growth. For this purpose, we used non-cancerous (Chang liver), cancerous (Hep3B, HepG2) and PXR overexpressing cancerous (human HepXR, mouse HepXR) cell lines.

**Fig 5:**
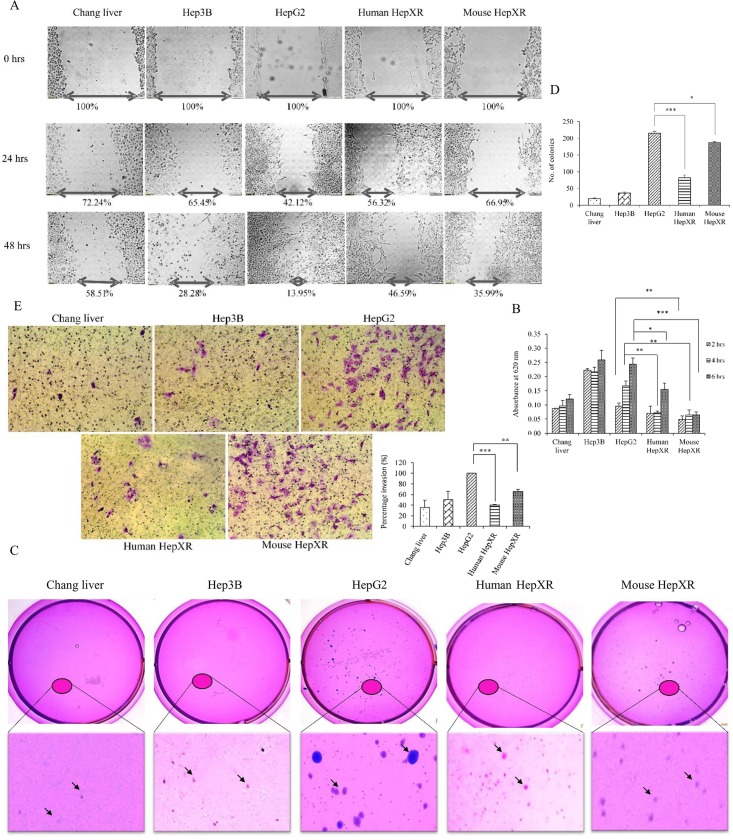
Human HepXR and mouse HepXR cells stably expressing higher levels of PXR exhibit reduced tumorigenic potential. All the experiments were performed with cell lines that included Chang liver, Hep3B, HepG2, human HepXR and mouse HepXR cells as described under ‘Materials and Methods’. A. In ‘wound healing assay’ cell lines were cultured to achieve 70–80% confluency. A scratch was inflicted with a 200 μl of pipette tip to create the wound. The width of the cell-free zone in the wound was monitored at 0 hrs, 24 hrs and 48 hrs and the migration ability of the cells to fill the wound gap was calculated using Image J software. Values presented are percentage of the wound gap as compared to control (100%). B. In ‘adhesion assay’ cell lines were seeded at 4 × 10^4^ cells per well in a 24-well plate. Cells were harvested at 2 hrs, 4 hrs and 6 hrs after fixing with 4% para-formaldehyde for 10 minutes followed by staining with 0.1% (W/V) crystal violet for 20 minutes. This was followed by solubilization in 1% Triton X 100 for 24 hrs and the adhesion ability of the cells was calculated by taking the spectrophotometric readings at 620 nm. Values presented are absorbance at 620 nm. The measured colour intensity was proportional to the number of adhered cells. C. In ‘colony formation assay’ the experimental cell lines were seeded with 5,000 cells with 0.35% agarose-DMEM per 35 mm plate after plating the 0.5% agarose-DMEM bottom layer as described under ‘Materials and Methods’. Plates were incubated at 37°C in humidified incubator and intermittently observed for 3 weeks. Plates were stained with 0.005% (W/V) crystal violet for 3–4 hrs followed by washing with PBS. Stained colony images were recorded with Sony BionZ X camera. Anchorage-independent growth of the cells was calculated by manually counting the colonies (indicated by arrows) using Microsoft office picture manager software at 200% zoom after randomly choosing the areas on the plate. D. In ‘cell invasion assay’ after seeding cell lines as mentioned in ‘Materials and Methods’, cultures were allowed to invade through the matrigel up to 36 hrs. Invaded cells were stained with crystal violet and counted to quantify. Values presented in the graph are number of colonies of cell-specific type in each plate. All the values are represented as the mean ±SE. The P-value represents the significance in human HepXR and mouse HepXR cell lines as compared to the control HepG2 cell line in all experiments. P-value <0.05 represented by a single asterisk (*), P-value <0.005 represented by a double asterisk (**), P-value <0.0005 represented by a triple asterisk (***).

In wound healing assay we found that human HepXR and mouse HepXR cells, which stably express human PXR and mouse PXR respectively, showed reduced cell migration to cover the inflicted wound after 24 hrs and 48 hrs time point as compared to the HepG2 cells (i.e. 56.32% and 46.59% in human HepXR, 66.95% and 35.99% in mouse HepXR and 42.12% and 13.95% in HepG2, Fig 5A). Also, human HepXR and mouse HepXR cell lines appeared to adhere less on the culture plate surface. Further, we performed cell adhesion assay with these cell lines in complete media onto the surface of 24-well plates at different time points. We observed that the number of human HepXR cells sticking onto the plate surface in 4 hrs and 6 hrs were about 2.3 and 1.62 fold less respectively, as compared with HepG2 cells (Fig 5B). Similarly, in case of mouse HepXR at 2 hrs, 4 hrs, 6 hrs time period the cells exhibited 1.9, 2.6 and 3.6 fold less adherence respectively, as compared with HepG2 cells (Fig 5B). To examine the tumorigenic index of the stable clone of human HepXR and mouse HepXR, we performed a ‘soft agar colony formation assay’. We found a significant decrease in the colony count with human HepXR cells (83 ± 15 colonies) and mouse HepXR cells (187 ± 7 colonies) as compared with HepG2 cells (216 ± 12 colonies, Fig 5C). To test the effect of PXR on the invasive property of a cancer cell, an invasion assay using the same cell lines was performed. We observed that percentage invasion of human HepXR and mouse HepXR cells were less (i. e. 40.2% and 65.9%) as compared to the HepG2 cells (Fig 5D).

### PXR reduces cell-ECM interaction in hepatic cancer cells

**Fig 6:**
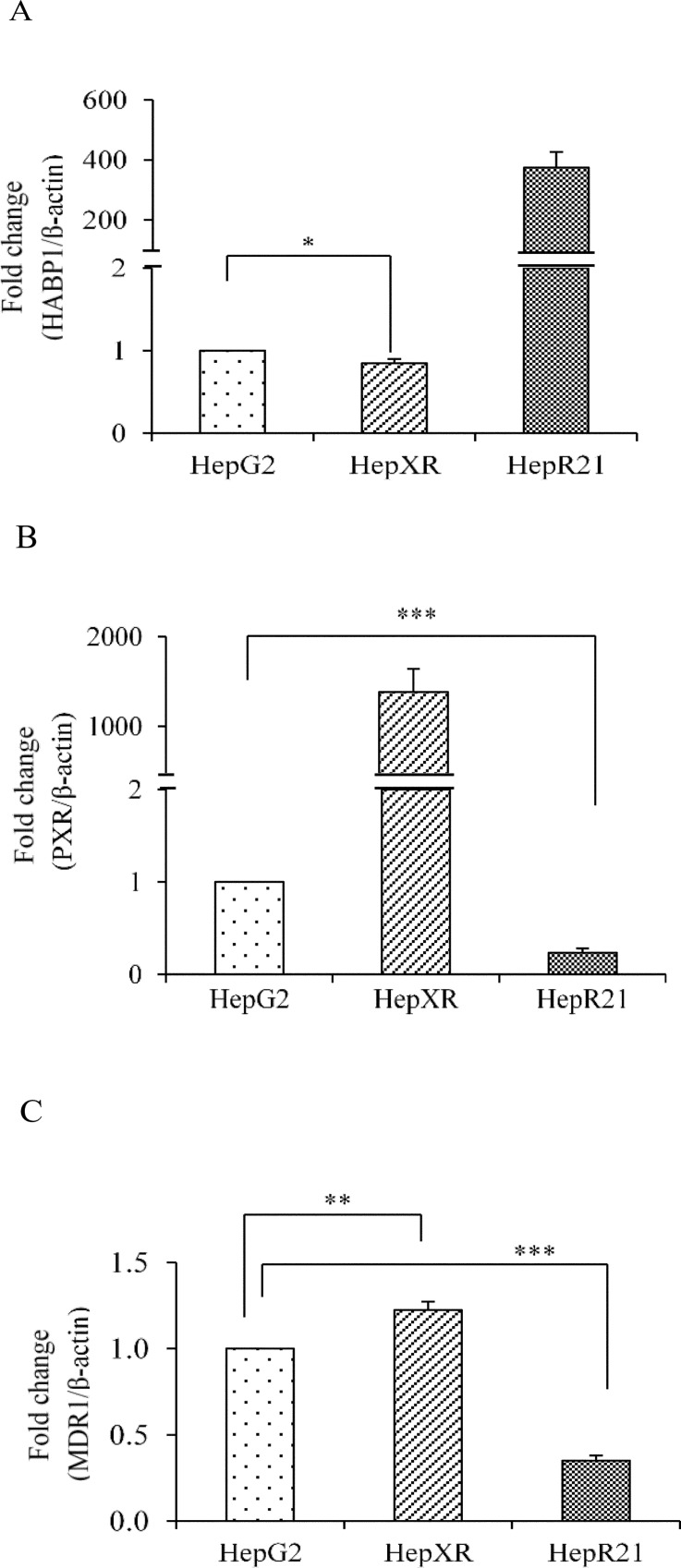
PXR reduces cell-ECM interactions in hepatic cancer cells. Total RNA was extracted from HepG2, human HepXR and HepR21 cell lines and used for real-time PCR analysis of A. human HABP1; B. human PXR; and C. human MDR1. β-Actin served as an internal control. The values are represented as the mean ±SE. The P-value represents the significance in human HepXR and HepR21 cell lines as compared to the control HepG2 cell line. P-value <0.05 represented by a single asterisk (*), P-value <0.005 represented by a double asterisk (**), P-value <0.0005 represented by a triple asterisk (***).

In the tumor, various cell types interact with each other and their micro-environment by exchanging information through cell-cell and cell-extracellular matrix (ECM) interactions. Hyaluronan (HA) is a multi-functional polysaccharide that is present in the extracellular and pericellular matrices of most tissues. Previously, we have shown that overexpression of HABP1 in HepG2 cells (HepR21 cell line) leads to enhanced cell survival and tumorigenicity by activating HA-mediated cell survival pathways [25]. In this study, the transcript level of PXR and HABP1 in human HepXR and HepR21 cells were compared using HepG2 cells as control. The results indicated that *PXR* suppresses *HABP1* by 0.16 fold (Fig 6A) while HABP1 suppresses PXR by 0.77 fold (Fig 6B). Moreover, to validate this observation we examined the expression of PXR-regulated gene *MDR1* in HepR21 and HepXR cells. We found a significant down-regulation of *MDR1* by 0.65 fold in HepR21 and 1.22 fold up-regulation of *MDR1* in HepXR cells. This suggests that PXR may reduce cell-ECM interaction in hepatic cancer (Fig 6C).

### Increase in intracellular PXR levels impedes cell proliferation while increases cell survival in hepatic cancer

**Fig 7:**
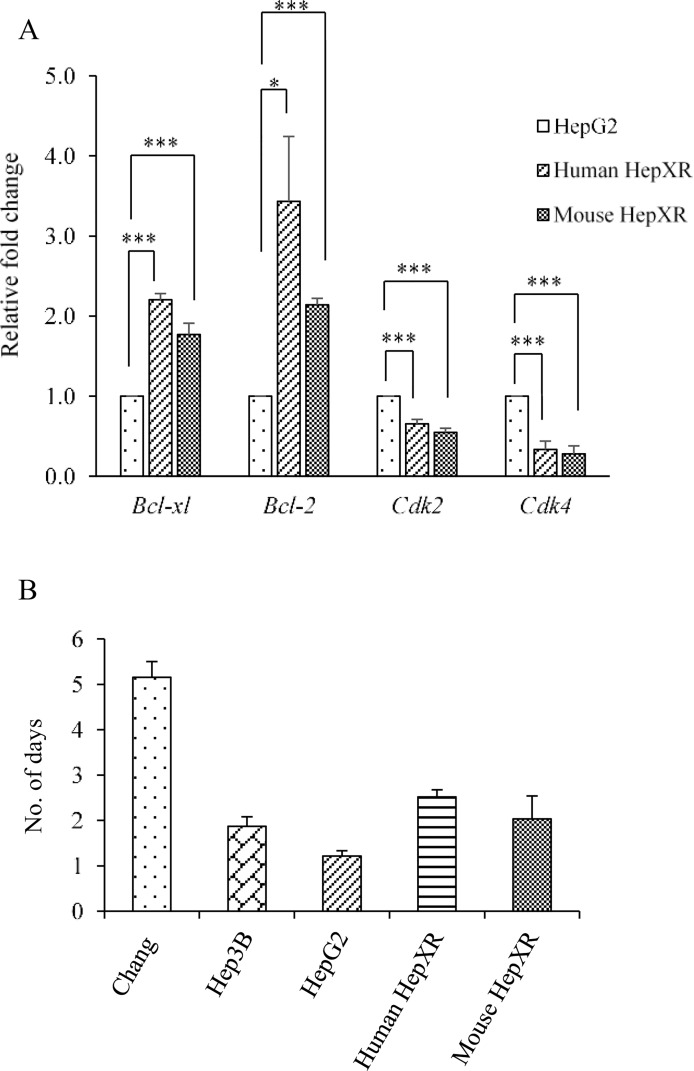
PXR enhances expression of apoptotic genes while reduces expression of cell-cycle regulatory genes in hepatic cancer cells A. Real-time PCR analysis was performed for *Bcl-xl*, *Bcl-2*, *Cdk2* and *Cdk4* with total RNA extracted from HepG2, human HepXR and mouse HepXR cell lines for various cell cycle and apoptosis regulatory genes. β-Actin served as an internal control. B. Higher cellular expression of PXR increased the doubling time in human HepXR and mouse HepXR cells in comparison to HepG2 cells. The experiments were performed three times with three samples of each cell lines and the values are represented as the mean ±SE. The P-value represents the significance in human HepXR and mouse HepXR cell lines as compared to the control HepG2 cell line. P-value <0.05 represented by a single asterisk (*), P-value <0.005 represented by a double asterisk (**), P-value <0.0005 represented by a triple asterisk (***).

It was observed that increased expression of PXR results into an increase in doubling time in human HepXR (2.5 days) and mouse HepXR (2.0 days) cells which is 1.21 days in HepG2 cells (Fig 7B). So, we examined the expression of cell cycle regulatory and apoptotic genes. Cyclin-dependent kinases (CDKs) are family of protein kinases having important roles in regulating the cell cycle through binding to regulatory proteins called ‘cyclins’. Our results showed that PXR reduces the expression of *CDK2* mRNAs in human HepXR and mouse HepXR cells to 0.65 and 0.54 fold, respectively as compared to the HepG2 cells (Fig 7A). Similarly, in comparison to HepG2 cells, reduction in the expression of *CDK4* mRNA was observed in human HepXR and mouse HepXR cells to 0.33 and 0.27 fold, respectively. The expression of mRNAs encoded by genes involved in apoptosis, *Bcl-2* and *Bcl-xL* were increased in human HepXR and mouse HepXR cells. The expression of *Bcl-xl* was enhanced in human HepXR and mouse HepXR cells by 2.20 and 1.76 fold, respectively as compared to the HepG2 cells (Fig 7A). Similarly, as compared to HepG2 cells, enhanced expression of *Bcl-2* mRNA was observed in human HepXR and mouse HepXR cells by 3.4 and 2.1 fold, respectively.

### *In vivo* overexpression of PXR does not contribute to hepatocarcinogenesis

**Fig 8:**
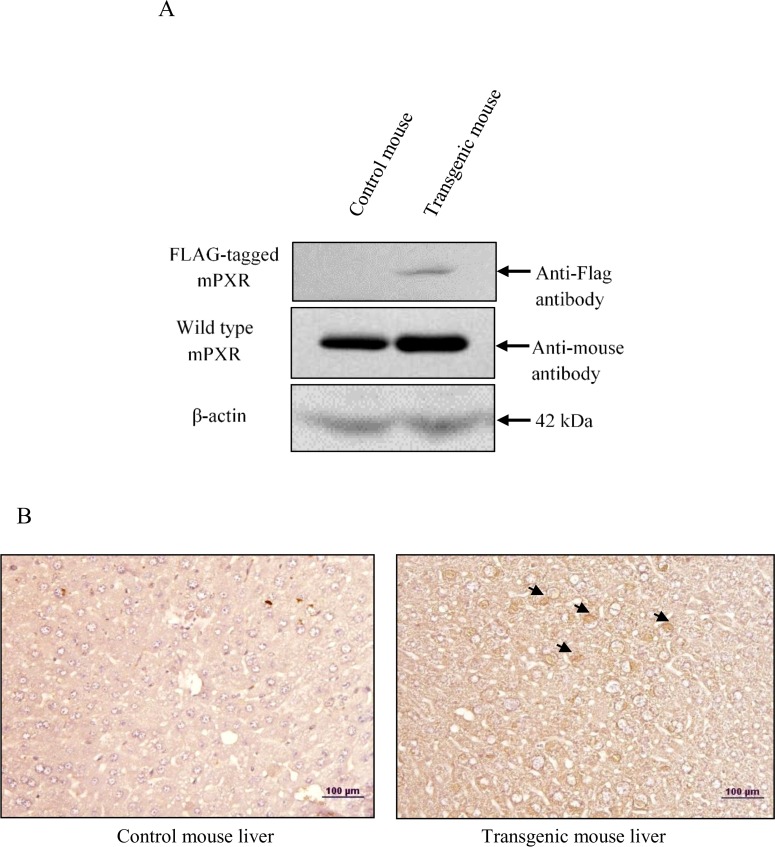
Overexpression of FLAG-tagged mPXR gene at protein level and its effect on tissue histology. A. Liver tissue extracts of Control and transgenic mice were electrophoresed (50 μg per sample) on a 10% SDS-PAGE. The proteins were transferred onto the methanol-activated PVDF membrane and probed with anti-FLAG antibody and anti-mouse PXR antibody (upper panels). β-Actin antibody served as control (lower panel). B. Control and transgenic mice were sacrificed and their livers were removed. Paraffin sections were prepared from control and transgenic mice livers harvested in the experiment and immunodetection were performed with anti-FLAG antibody (indicated by arrows). The immunodetected tissues were viewed and recorded at 20X magnification using a light microscope. The experiments were performed with three samples of each of the controls and the transgenic mice.

After examining the potential role of PXR on various tumorigenic properties in cancer cell lines, we further made a histological examination of PXR overexpression *in vivo*. For this, we generated transgenic mice expressing FLAG-tagged mouse PXR and confirmed the expression of transgene at mRNA and also protein level by western blot (Fig 8A) and immunohistochemistry (Fig 8B). We observed that overexpression of PXR does not alter the histological profile that would be suggestive of hepatic cancer (i.e. hepatic cord thickening and hepatic portal triad disruption etc.) as were observed in DEN-induced hepatic cancer tissues (Figure A in S1 File). This implies that under normal physiological conditions higher expression of PXR does not appear to contribute to hepatocarcinogenesis.

## Discussion

Previous reports indicate that altered expression of PXR in cancer is dependent upon tissue type and tissue micro-environment [3–13]. A higher expression of PXR in breast [7], esophageal [11] and bone [13] cancers is associated with cancer progression by multidrug resistance. However, lower expression of PXR in prostate [9], colon [5] and cervical [14] cancer is associated with cancer suppression while, in colon [6] and endometrial [10] cancer differential expression of PXR is reported. In hepatic cancer PXR function is still poorly defined. To examine whether and how PXR could be involved in hepatic carcinogenesis, we analyzed the expression of PXR in DEN-induced hepatic cancer tissues. We found that PXR is significantly down-regulated in DEN-induced hepatic cancer (Fig 1). Based on this evidence, we hypothesized that PXR may not be significantly involved in hepatic carcinogenesis or in cancer progression. Simultaneously, we examined the expression of CAR (another closely related xenobiotic receptor in hepatic cancer tissues) as it regulates some of the overlapping sets of genes with PXR. However, we did not find significant downregulation of this receptor. Further, RXR-α, a heterodimeric partner of PXR and CAR, was found to be significantly upregulated in hepatic cancer tissues (Fig 1). Overall, the observations prompted us to speculate that PXR may be exerting negative effects on hepatic cancer progression.

Next, the expression profiles of phase I, II DMEs and drug transporters that are regulated by PXR were evaluated in hepatic cancer tissues and compared with the normal controls. Representative PXR target genes from each phase of detoxification machinery were chosen on the basis of their established functions in detoxification. Our results showed significant down-regulation of phase I (e.g. CYP3A11), phase II (e.g. GSTa2) DMEs and upregulation of drug transporters (e.g. MDR1 and MRP3) in hepatic cancer tissues (Fig 2 and Figure B in S1 File). Such downregulation of PXR, CYP3A11 and GSTa2 and upregulation of drug transporters is expected to have important implications in the treatment regimen of hepatic cancer that could potentially dysregulate hepatic drug biotransformation and bioavailability of administered drugs. We further explored the possible cause of downregulation for PXR and its target genes. Interestingly, in context to human obstructive cholestasis down-regulation of Phase II DMEs and upregulation of drug transporters is reported to be executed by an inflammatory cytokine TNF-α [27–29]. LPS-induced cytokines IL-1β, IL-6, and TNFα, which are also expressed in inflammatory bowel disease, are reported to down-regulate PXR and CYP genes *in vivo* [17, 30–33]. Furthermore, NF-κB and PXR upon activation mutually repress each other’s expression [18, 34, 35]. Considering that hepatic cancer is an inflammation-related cancer we attempted to examine the role of inflammatory proteins in downregulation of PXR and its target genes. In this direction, we examined the expression of inflammatory proteins IL6, STAT3, TNF-α and NF-κB in hepatic cancer tissues. Interestingly, when compare to normal we observed their higher expression in hepatic cancer tissues (Fig 3).

Our observations revealed that lower expression of PXR, CYP3A11 and GSTa2 are inversely correlated with higher expression levels of inflammatory proteins like IL6, TNF-α and NF-κB both at transcript and protein levels (Fig 4 and Figure C in S1 File). The observed decrease in the levels of PXR and its target genes represents the influence of inflammation in liver and expression of genes important in drug metabolism and disposition in hepatic cancer. This implies that onset of appearance of inflammatory proteins have the potential to influence the drug metabolizing capacity in hepatic cancer.

From our *in vivo* experiments, it appeared that exclusive down-regulation of PXR in hepatic cancer may not contribute to progression of hepatic cancer. Therefore, to further examine this hypothesis, we performed some critical cell culture assays using a hepatic cancer cell line stably overexpressing PXR. We found that higher expression levels of PXR reduces tumorigenic potential by reducing cell migration (from wound healing assay), cell adhesion (from cell adhesion assay), anchorage-independent growth (from colony formation assay) and cell invasion (from cell invasion assay) of hepatic cancer cells (Fig 5). Our results suggested that expression of PXR in advanced hepatic tumor may help to reduce the migration of cancer cells by reducing invasion in the surrounding tissues. This reduces neoplastic cells to enter lymphatic and blood vessels for dissemination into the circulation followed by further reduction to their attachment and anchorage-independent metastatic growth in distant organs. Though, the overall observations are supportive of protective/defensive role of PXR, an *in vivo* investigation to explore molecular mechanisms involved these processes are needed.

Previously, our laboratory has shown that elevated expression of hyaluronan-binding protein 1 (HABP1), an important component of extracellular matrix enhances tumorigenicity in HepG2 cells [25]. The interaction between cancer cells and their niche is known to play an important role in cancer development. Interestingly, our data show that PXR and HABP1 mutually repress each other's expression (Fig 6). This observation suggests that higher PXR level reduces interaction of cancer cell with its surrounding microenvironment that may reduce carcinogenesis. There are reports which showed that PXR is involved in the proliferation of diverse human cancers, and is associated with induction of cell proliferation [3, 7, 11]. On the contrary, PXR is also reported to suppress proliferation of breast and colon cancer cell lines and also tumorigenicity in colon xenograft models [5, 19]. We examined the cell proliferation of PXR overexpressing cell lines by measuring the doubling times, which was observed to be enhanced (Fig 7B). To decipher the possible mechanism for reduced cell proliferation, we examined the expression profile of cell cycle regulatory and apoptotic genes. Our assessment of the expression levels of cell cycle regulatory genes *CDK2* and *CDK4* in PXR over-expressed cell lines exhibited their down-regulation (Fig 7A). These results implied that PXR may also be controlling the tumour cell proliferation. Additionally, we examined the expression of anti-apoptotic genes *Bcl-2* and *Bcl-xl*. We observed enhanced expression levels of *Bcl-2* and *Bcl-xl* genes that may lead to increased cell survival in hepatic cancer cells. This may be an ‘adaptive response’ attenuating hepatic cancer (Fig 7A). Overall, PXR appears to be in a protective role in hepatic cancer. To further confirm its protective role in hepatocarcinogenesis, we overexpressed the FLAG-tagged mouse PXR *in vivo* in transgenic mice. We did not observe alteration in the histological profile (i.e. hepatic cord thickening and hepatic portal triad disruption etc.) in the liver tissue of transgenic mice (Fig 8B) which were observed in DEN-induced hepatic cancer tissue (Figure A in S1 File). This suggests that under normal physiological condition, higher expression of PXR does not contribute to symptoms suggestive of hepatocarcinogenesis.

*In vitro* PXR overexpression in hepatic cancer cell line showed MDR1 upregulation implying that it is a PXR-regulated transporter. However, *Mdr1* and *Mrp3* were also found upregulated *in vivo* while PXR is downregulated suggesting that under hepatic cancerous condition *Mdr1* and *Mrp3* may be regulated by some other pathways and this may be associated with higher levels of inflammatory proteins or other nuclear receptors, as is reported in obstructive cholestasis [28]. The underlying mechanisms for this upregulation of *Mdr1* and *Mrp3* genes in cancerous condition need to be investigated further. Also, there are some studies showing that chemical compounds like phenobarbital and fluopyram activated PXR and suggested the potential role of PXR in hepatic cancer progression [36, 37]. However, our study differs from these since our objective was to examine the levels of PXR and its target genes in absence of influence of any exogenous chemical/ligand. We used only a single dose of DEN to induce hepatic cancer in mice, nine months before sacrifice. So, our results showed the absolute endogenous levels of PXR and its target genes in hepatic cancer which are likely to be influenced by inflammatory proteins.

Herein, the study provides an important evidence where a complex interplay between inflammatory proteins with PXR and its target gene CYP3A11 and GSTa2 are associated with impaired hepatic detoxification in hepatic cancer. Overall, levels of PXR and its target genes were found to be down-regulated in hepatic cancer. This downregulation conceivably contributed to the impaired drug metabolism which is a consequence of enhanced levels of inflammatory proteins. On the other hand, higher expression levels of PXR reduces the tumorigenic potential of hepatic cancer cells and is not involved in hepatocarcinogenesis under normal physiological condition *in vivo*.

Our observations provide a basis for the prospective studies that may define possible mechanisms to reveal how PXR reduces hepatocarcinogenesis *in vivo*. These observations provide an understanding in hepatic cancer for the questions about expression levels of PXR, its influences on the drug detoxification capacity and tumorigenic potential.

## Supporting Information

S1 File Supporting figures and table for the present study. (DOC)
