# Supplementary material for: Role of PXR in Hepatic Cancer: Its Influences on Liver Detoxification Capacity and Cancer Progression
Source: PLoS One. 2016 Oct 19;11(10):e0164087. doi: 10.1371/journal.pone.0164087 (PMC5070842; doi:10.1371/journal.pone.0164087)
Supplement: S1 File — (DOC) [file pone.0164087.s001.doc]

**SUPPORTING DATA**

**SUPPORTING FIGURE LEGENDS**

**SUPPORTING FIGURE A**

A

B

**Supporting Figure A. Histopathological evaluation and expression of AFP in DEN-induced hepatic cancer. A.** Mice were sacrificed after 9 months of introducing the DEN and liver removed as shown in the figure (tumor was indicated by blank arrows). Paraffin sections were prepared from normal saline-treated control and DEN-induced hepatic cancer tissues. The tissue sections were stained with hematoxylin and eosin and viewed at 20X magnification. Histopathological evaluation showed hepatic cord thickening and hepatic portal triad disruption (as indicated by filled arrows).**B.** Total RNA was extracted from mice liver tissues of normal saline- treated control and DEN-treated mice and used for PCR analysis of AFP (112 bp). Lower panel shows GAPDH that served as an internal control. Samples were examined on 2% agarose gel. Marker = Marker 50 bp DNA ladder. **C.** Typical expression of AFP, as shown in panel B, was quantified by using image J software. **D.** Cell extracts of hepatic cancerous mice and control mice liver tissues were electrophoresed (50 µg per sample) on a 10% SDS-PAGE and western blot analysis was performed. The proteins were transferred to methanol-activated PVDF membrane and probed with anti-mouse AFP antibody (upper panel). β-Actin served as control (lower panel); Lanes 1 and 2, correspond to normal saline treated control mice; Lane 3 and 4, correspond to DEN-induced hepatic cancerous mice. **E.** The relative endogenous AFP expression in control and DEN-induced hepatic cancer mice was quantified by densitometry. The experiments were performed with six samples of each control and DEN-induced cancerous mice and the values are represented as the mean ±SE. The P-value represents the significance in DEN-induced hepatic cancer mice as compared to the control mice. P-value <0.05 represented by a single asterisk (*), P-value <0.005 represented by a double asterisk (**), P-value <0.0005 represented by a triple asterisk (***).

**Supporting figure B**

**Supporting Figure B. Enhanced expression of MDR1 in DEN-induced hepatic cancer.** Total RNA was extracted from mice liver tissues of normal saline-treated control and DEN-treated mice and used for PCR analysis of MDR1 (564 bp). As compared to control a high level of MDR1 is observed. Lower panel shows GAPDH that served as an internal control. Samples were examined on a 2% agarose gel.

**Supporting figure C**

**Supporting Figure C. Correlations between the reduced transcript levels of PXR and PXR-regulated Phase I, Phase II DMEs and enhanced transcript levels of inflammatory genes in hepatic cancer**. Relative mRNA levels of TNF-α and P65 were correlated with PXR, CYP3A11 and GSTa2 mRNA levels using Pearson’s correlation coefficient (r) in scattered plot. Respective P-value represents the significance between the correlations.

**SupportingTable: A**

| **Primer** | **Sequences** | **Annealing Temp.(˚C)** | **Product size (bp)** |
| --- | --- | --- | --- |
| Mouse AFP | 5’-GCT ACC ATC ACC TTT ACC CAG-3’  5’- TTC TAA ACA CCC ATC GCC AG-3’ | 60 | 112 |
| Mouse GAPDH | 5’-CTC ATG ACC ACA GTC CAT GC-3’  5’-CAC ATT GGG GGT AGG AAC AC-3’ | 60 | 201 |
| Mouse PXR | 5’-GGA AGA GCC CAT CAA CGT AG-3’  5’-TGC ATC CTT CAC ACG TCA T-3’ | 55 | 110 |
| Mouse RXR | 5’-TCC TTC ACC AAG CAC ATC TG-3’  5’-TCT TTG CGT ACT GTC CTC TTG-3’ | 60 | 113 |
| Mouse GSTa2 | 5’-GGG TGG AGT TTG AAG AGA AG-3’  5-‘TGG CGA TGT AGT TGA GAA TG-3’ | 60 | 150 |
| Mouse MRP3 | 5’-ACG CCA TCA CCA TAC ACA AC-3’  5’-GTA AGG CAG ACA CCA GAG AAG A-3’ | 60 | 149 |
| Mouse STAT3 | 5’-GGA GCA GAG ATG TGG GAA TG-3’  5’-GTG GGT CTC TAG GTC AAT CTT G-3’ | 60 | 130 |
| Mouse Rel-A (P65) | 5’-CGC AGT ATC CAT AGC TTC CAG-3’  5’-ATT CAA GTC ATA GTC CCC GC-3’ | 60 | 144 |
| Mouse TNF-α | 5’-GTT GTA CCT TGT CTA CTC CCA GG-3’  5’- GGT TGA CTT TCT CCT GGT ATG AG-3’ | 60 | 130 |
| Mouse MDR1  (For RT-PCR) | 5’-ATG CAG ATT GGC TGG ACA AGC-3’  5’-CAG ATG ACA ATC CAA TAA CAG-3’ | 64 | 564 |
| Mouse CAR | 5’-GGA GCG GCT GTG GAA ATA TTG-3’  5’-TCC ATC TTG TAG CAA AGA GGC C-3’ | 60 | 150 |
| Mouse CYP3A11 | 5’-CAA GGG TTT ATG GAA ATT CGA-3’  5’-TAA TCG TCT CTG GGT CTG TGA CA-3’ | 60 | 110 |
| Human PXR | 5’-GAC ATG TGA AGG ATG CAA GG-3’  5’- CTC TCC AGG CAC TTG CGC A-3’ | 60 | 147 |
| Human β-actin | 5’- GTG GTG GTG AAG CTG TAG CC-3’  5’-CCA CAC TGT GCC CAT CTA CG-3’ | 60 | 129 |
| Human HABP1 | 5’- ATC AAC TCC CAA TTT CGT GGT T-3’  5’- TCC TCT GGA TAA TGA CAG TCC AA-3’ | 60 | 84 |
